# Comparative analysis of microbiota along the length of the gastrointestinal tract of two tree squirrel species (*Sciurus aberti* and *S. niger*) living in sympatry

**DOI:** 10.1002/ece3.5789

**Published:** 2019-11-11

**Authors:** Andrew Reed, Jon C. Pigage, Helen K. Pigage, Cody Glickman, Jeremy M. Bono

**Affiliations:** ^1^ Biology Department University of Colorado Colorado Springs Colorado Springs CO USA; ^2^ Computational Bioscience Graduate Program University of Colorado Denver Anschutz Medical Campus Aurora CO USA

**Keywords:** *alpha*‐diversity, *beta*‐diversity, cellulose digestion, fiber digestion, microbiome, *Prevotella*

## Abstract

Microbiota inhabiting the gastrointestinal (GI) tract of animals has important impacts on many host physiological processes. Although host diet is a major factor influencing the composition of the gut micro‐organismal community, few comparative studies have considered how differences in diet influence community composition across the length of the GI tract. We used 16S sequencing to compare the microbiota along the length of the GI tract in Abert's (*Sciurus aberti*) and fox squirrels (*S. niger*) living in the same habitat. While fox squirrels are generalist omnivores, the diet of Abert's squirrels is unusually high in plant fiber, particularly in winter when they extensively consume fiber‐rich inner bark of ponderosa pine (*Pinus ponderosa*). Consistent with previous studies, microbiota of the upper GI tract of both species consisted primarily of facultative anaerobes and was less diverse than that of the lower GI tract, which included mainly obligate anaerobes. While we found relatively little differentiation between the species in the microbiota of the upper GI tract, the community composition of the lower GI tract was clearly delineated. Notably, the Abert's squirrel lower GI community was more stable in composition and enriched for microbes that play a role in the degradation of plant fiber. In contrast, overall microbial diversity was higher in fox squirrels. We hypothesize that these disparities reflect differences in diet quality and diet breadth between the species.

## Graphical abstract

In this study, we compare the microbiota along the length of the gastrointestinal tract of two tree squirrels living in sympatry.

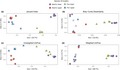

## 1 INTRODUCTION

Animals harbor complex communities of microorganisms within their gastrointestinal (GI) tracts. These communities have an important impact on their host through influences on essential processes, such as nutrient acquisition, immunity, development, and behavior (Lathrop et al., 2011; Ley, Lozupone, Hamady, Knight, & Gordon, 2008; Sommer & Bäckhed, 2013; Tremaroli & Bäckhed, 2012). Comparative studies within and between species have revealed considerable variation in the microbiome, and understanding the factors that contribute to this variation is important for understanding how these communities influence host ecology and evolution (Ley, Lozupone, et al., 2008; Sharpton, 2018).

In mammals, host diet is an important driver of the community composition of the gut microbiota (Ley, Lozupone, et al., 2008; Muegge et al., 2011; Tremaroli & Bäckhed, 2012). For example, comparative studies have shown that mammalian gut microbial communities generally cluster according to feeding strategy (e.g., carnivores vs. omnivores vs. herbivores) and, within a single species, individuals with different diets often have distinct microbiota (David et al., 2014; Ley, Hamady, et al., 2008; Muegge et al., 2011). The microbial community of the GI tract is especially important for herbivorous mammals, as it is required to break down indigestible plant fiber and convert it to energy useable by the host and may also help metabolize toxic plant secondary compounds (PSCs) (Kohl, Miller, Marvin, Mackie, & Dearing, 2014; Kohl, Weiss, Cox, Dale, & Dearing, 2014; Mackie, 2002). Plant‐feeding mammals typically have a more diverse microbiota than their carnivorous relatives and often have enlarged and/or elongated digestive tracts to facilitate fermentation of plant material by the resident microbial community (Ley, Hamady, et al., 2008).

Most comparative microbiome studies have focused on fecal samples, which are not necessarily representative of the microbiome across all regions of the gastrointestinal tract (Hillman, Lu, Yao, & Nakatsu, 2017). Factors such as pH, oxygen availability, and production of antimicrobial immune products vary along the length of the tract, and regions of the tract are functionally distinct (Donaldson, Lee, & Mazmanian, 2016). As expected, studies that have analyzed the microbiome of different gut regions have found considerable variation even within individuals (Gu et al., 2013; Kohl, Brun, et al., 2017; Kohl, Dearing, & Bordenstein, 2017; Kohl, Miller, et al., 2014; Lu et al., 2015; Suzuki & Nachman, 2016), but few studies have done this in a comparative framework across species.

The eastern fox squirrel (*Sciurus niger*) and Abert's squirrel (*Sciurus aberti*) are tree squirrels that co‐occur in a narrow area of sympatry along the Front Range of Colorado, USA, and some areas of New Mexico (Geluso, 2004; Hoover & Yeager, 1953). Fox squirrels are generalist omnivores, consuming a variety of items such as fruits, grains, seeds, buds, nuts, tubers, insects, and bird eggs (Lechleitner, 1969). As scatter hoarders, fox squirrels also cache food for consumption during winter months when favored food items would not otherwise be available (Delgado & Jacobs, 2017). Abert's squirrels are found almost exclusively in association with ponderosa pine forests (Allred, 2010), although they have persisted in other conifer forests following introductions by humans (Derbridge & Koprowski, 2019; Edelman & Koprowski, 2005). In their native range, the diet of Abert's squirrels consists mainly of food items related to ponderosa pine, including inner bark (phloem), apical buds, seeds, cones, pollen strobili, dwarf mistletoe, and fungi associated with tree root systems. Other miscellaneous items (e.g., acorns) often make up a smaller portion of their diet (Allred, 2010; Hall, 1981; Keith, 1965; States & Wettstein, 1998). The diet varies seasonally depending on availability of food items, with inner bark, and fungi being the only food items consumed throughout all months of the year (Allred, 2010; Keith, 1965). Although Abert's squirrels are known to occasionally cache scattered food items, they are particularly dependent on inner bark in the winter, with several past studies demonstrating that this food resource constitutes the majority of the diet in winter months (ranging from approximately 60% to over 90% of the overall diet) (Allred, 2010; Hall, 1981; Keith, 1965; States & Wettstein, 1998). This is a low‐quality food source, lacking in available nitrogen (<1%) and readily digestible nonstructural carbohydrates (4%–6%). Moreover, it is relatively high in toxic polyphenols, and with a consistency resembling sawdust or wood shavings, it is rich in cellulose, lignin, and other plant structural elements that are not readily digestible by mammals (Snyder, 1992). Consistent with expectations based on this low‐quality, high‐fiber diet, Abert's squirrels have a highly elongated GI tract, creating more surface area for digestion to occur and presumably decreasing transit time of food through the gut (Murphy & Linhart, 1999). In a comparative study, the overall surface area of the Abert's squirrel GI tract was 61% larger than that of the fox squirrel, with the cecum and large intestine being particularly enlarged (125% and 87% larger, respectively) (Murphy & Linhart, 1999).

Here, we use 16S amplicon sequencing to compare the microbiota along the length of the GI tract (stomach, small intestine, cecum, large intestine, and fecal material) both within and between fox and Abert's squirrels collected from the same study site in Colorado, USA. We predicted that within each species, different regions of the GI tract would harbor distinct microbial communities. Furthermore, based on differences in diet between fox and Abert's squirrels, we expected their gut microbial communities would be highly differentiated. Specifically, we predicted that the community inhabiting Abert's squirrels GI tracts would be enriched for taxa involved in the breakdown of plant structural compounds, such as cellulose and lignin, given their frequent use of inner bark as a food resource. Moreover, we predicted that these differences would be most evident in regions of the GI tract exhibiting the most morphological differentiation between the species (i.e., cecum and large intestine). We also compared the interindividual variation and overall diversity of the microbial communities associated with each squirrel species.

## 2 MATERIALS AND METHODS

### 2.1 Sample collection

All animals were trapped using Havahart live traps (60.96 cm × 17.78 cm × 17.78 cm) (Havahart) in a mixed ponderosa pine and Gambel oak (*Quercus gambelii*) forest from 15 March 2016 through 22 April 22, 2016 (elevation ~ 2,200 m). There was no snow cover at the time we began sampling, and no additional snow accumulated over the time window of the study. We selected this time window to control for seasonal variation in the gut microbiome and to sample Abert's squirrels at a time when previous studies have shown that they would be highly likely to consume inner bark (Hall, 1981; Keith, 1965; States & Wettstein, 1998). Indeed, we observed numerous clippings of terminal branches in trapping areas, and inner bark was observed in the stomach contents of sampled Abert's squirrels. Given the limited number of animals we could sample, we focused exclusively on females in order to control for additional sex‐dependent variation in the gut microbiome (male squirrels were released upon capture). Squirrels were euthanized via inhalation of isoflurane, and GI tract dissections were performed in an aseptic environment immediately following euthanasia. Once the GI tract was removed from the animal, incisions were made into the stomach, small intestine, cecum, and large intestine. For the stomach and cecum, we made a single incision and swabbed the inner walls of the structures with sterile swabs. Given the considerable length of the small and large intestine, we sampled from multiple locations along the length of these structures. For the small intestine, we divided the total length (pyloric valve to ileocecal junction) into thirds and swabbed the mid‐point of each section. Likewise, we divided the large intestine into halves and swabbed the mid‐point of each section. We also swabbed fecal material in the most distal section of the large intestine (heretofore referred to as the fecal sample). Individual swabs (*N* = 9 per individual) were immediately stored in separate Eppendorf tubes and maintained at −80°C until sample processing. All animals were trapped, handled, and euthanized following a protocol approved by the Institutional Animal Care and Use Committee (IACUC) (Protocol # UCCS‐16‐001) and Colorado Parks and Wildlife Scientific Collection License # 16TR2104.

### 2.2 DNA extraction and 16s rRNA gene sequencing

We used the MoBio Powersoil DNA Isolation Kit (QIAGEN) to extract DNA following the manufacturer's protocol. Sample concentration (ng/µl) and 260/280 ratios were measured using a Thermo Scientific NanoDrop 2000 Spectrophotometer (Thermo Fisher Scientific). Sequencing libraries of the V4 region (~250 bp) of 16S rRNA were prepared and sequenced by BGI Americas using the llumina MiSeq platform with 250 bp paired‐end reads.

### 2.3 Data analysis

We processed and analyzed the sequence data using plugins available in QUANTITATIVE INSIGHTS INTO MICROBIAL ECOLOGY 2 (QIIME 2) v. 2017.11.0 (Bolyen et al., 2018) and R v. 3.4.0. Due to low‐read counts, we removed seven small intestine samples from all analyses. These samples included only one of the three small intestine samples for two fox and two Abert's squirrels, and all three samples for an additional Abert's squirrel. After demultiplexing, we used the “dada2” Qiime 2 plugin to identify amplicon sequence variants (ASVs) (Callahan et al., 2016). Taxonomy was then assigned with the “classify‐sklearn” function of the “q2‐feature‐classifier plugin” using a pretrained Naïve Bayes Classifier trained on Silva 119 99% operational taxonomic units (OTUs) from 515F/806R region of 16S rRNA sequences (Bokulich et al., 2018). Sequences from mitochondria and chloroplasts were then filtered out, leaving a total of 5,190,422 sequences representing 2,455 taxonomic features across the 57 samples (range per sample: 14,886–153,760). We aligned sequences with MAFFT (Katoh & Standley, 2013) using the “alignment” plugin, and nonconserved and highly gapped regions were removed using the “mask” function. We built a phylogenetic tree with the “Fasttree” function (Price, Dehal, & Arkin, 2010) in the “phylogeny” plugin, which was rooted with the mid–point root function.

### 2.4 Diversity analyses

*Alpha*‐diversity analyses were carried out using the “diversity” plugin. Given the similarity among multiple samples taken from the small (three samples) and large intestines (two samples) within an individual, we merged samples from each region together in each individual using the “group” function of the “feature table” plugin (i.e., the three samples from the small intestine were combined, and the two samples from the large intestine were combined). We rarefied samples to a depth of 35,005 sequences as this corresponded to the sample with the smallest number of sequences in the dataset (Weiss et al., 2017). We assessed *alpha*‐diversity with three different metrics: number of ASVs, Shannon's *H* (Shannon & Weaver, 1949), and Faith's phylogenetic diversity (Faith, 1992). The number of ASVs is a measure of species richness that does not account for abundance, Shannon's *H* is a diversity estimate that accounts for abundance and evenness of taxa, while Faith's phylogenetic diversity incorporates phylogenetic distance between taxa into the measure of diversity. We analyzed *alpha*‐diversity estimates using the “lme4” (Bates, Mächler, Bolker, & Walker, 2015) and “lmerTest” (Kuznetsova, Brockhoff, & Christensen, 2017) packages in R. The mixed model included two main effects (species and sample location) and their interaction, and individual identity was included as a random factor to account for the fact that multiple samples were taken from each individual. The interaction term was not significant in any of the analyses, so we removed it and the models were run again with only the main effects.

We used four metrics to assess *beta*‐diversity: Jaccard index (Jaccard, 1908), Bray–Curtis dissimilarity (Weiss et al., 2017), unweighted UniFrac (Lozupone & Knight, 2005), and weighted UniFrac (Lozupone, Hamady, Kelley, & Knight, 2007). The Jaccard index considers only presence/absence, while Bray–Curtis dissimilarity also takes abundances into account. The two UniFrac metrics are both based on phylogenetic distances, with the weighted version also taking abundances into account while the unweighted version only considers presence/absence. We compared *beta‐*diversities using the PERMANOVA test implemented in “beta‐group‐significance” with significance assessed using 9999 permutations (Anderson, 2001). Since the implementation of this test does not allow for random effects that would account for multiple measurements taken from a single individual, we generated a new feature table for this analysis where all samples within an individual from the upper (stomach and small intestine) and lower (cecum, large intestine, and fecal) GI tract were merged (e.g., each individual had two samples representing upper and lower portions of the GI tract). We rarefied samples to a depth of 71,778 sequences, which was equal to the number of sequences in the samples with the fewest sequences (Weiss et al., 2017). PERMANOVA is typically used to compare centroid locations across groups, but can also be sensitive to differences in within‐group dispersion among samples (Anderson, 2001). We thus also tested for differences in levels of within‐group dispersion using the “betadisper” function in the “vegan” package (Oksanen et al., 2017) for R. Significance was assessed using the permutation procedure in “betadisper” (9999 permutations), and false‐discovery rate was controlled using the Benjamini–Hochberg correction (Benjamini & Hochberg, 1995). Distance matrices were also used for plotting principal coordinates analysis (PCoA), using the “phyloseq” package for R (McMurdie & Holmes, 2013).

### 2.5 Core microbiome and differential abundance analyses

To identify and compare core microbiomes of fox and Abert's squirrels, we used the “core‐features” function in the “feature‐table” plugin. We analyzed upper (small intestine and stomach) and lower (cecum, large intestine, fecal) GI tract samples separately given major differences in the microbiota present in these locations.

To identify taxa that were differentially abundant in comparisons of upper and lower GI tract samples within and between species, we used two methods: analysis of composition of microbiomes (ANCOM) (Mandal et al., 2015) and Gneiss (Morton et al., 2017). ANCOM is a method that accounts for the compositional data structure inherent to comparisons of microbial communities. Feature count data tables were exported to R without rarefying, and the analyses were conducted using the “ANCOM.main” function in the ANCOM 2.0 R package. The models for between species comparisons (upper and lower GI samples from the two species were compared separately) included the fixed main effect of species, and individual identity was coded as a random effect to account for multiple samples taken from each individual. For comparisons of upper and lower GI samples within species, the number of individuals was reduced to four, which is below generally recommended guidelines for estimation of variance associated with random effects. As a solution, along with a fixed effect for GI region, we also included individual id as a fixed effect in these models. This method still accounts for individual variation while testing for the differences between upper and lower GI samples, though it may have reduced power compared to the alternative approach of treating individual id as a random effect. In both within and between species analyses, a taxa‐wise multiple testing correction was applied with a significance cutoff of 0.05. Any ASVs with proportion of zeroes greater than 90% were excluded from the analysis. We analyzed lists of differentially abundant taxa produced by these tests using hypergeometric tests to determine whether any particular taxa were enriched (i.e., overrepresented in a sample relative to frequency in the overall dataset).

Given the challenges associated with analyzing differential abundances when data are compositional, Gneiss uses the concept of balance trees to identify subcommunities within the microbiota that shift between groups rather than identifying individual features that are differentially abundant (Morton et al., 2017). We used the “Gneiss” plugin to perform comparisons within and between species as described above (samples were not rarefied prior to analysis). Only ASVs present in at least two samples were retained for these analyses. Balance trees were constructed using correlation clustering, which uses a distance measure to cluster together microbes that tend to co‐occur in the same samples. The first 10 balances (y0–y9) for each analysis were exported and analyzed in R, as lower‐order balances typically explain the most variation. For between species comparisons of upper and lower GI samples, we used “lme4” (Bates et al., 2015) and “lmerTest” (Kuznetsova et al., 2017) packages. The mixed‐effects model included species as a fixed main effect, and individual id was included as a random effect to account for multiple sampling from the same individuals. For within species comparisons between upper and lower GI samples, we used the “lm” function to analyze linear models that included GI region and individual as fixed effects (see above). In all cases, significance of GI region was assessed after applying the Benjamini–Hochberg false‐discovery rate correction (Benjamini & Hochberg, 1995).

## 3 RESULTS

**Figure 1:**
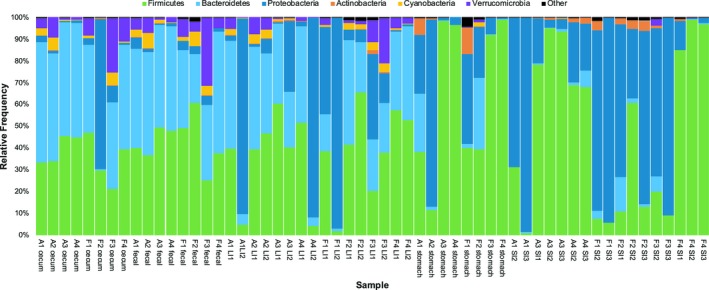
Relative frequency of bacterial phyla in samples from GI tracts of Abert's and fox squirrels. For each sample, the species is denoted by an “A” for Abert's and an “F” for fox. Each individual is denoted by a number (1–4), and multiple samples from the same individual/region are designated by numbers. LI, large intestine; SI, small intestine

Overall, gut microbiomes included representatives from 25 phyla of Bacteria and two phyla from Archaea (Table S1). Most of these taxa were rare, however, with only six Bacterial phyla accounting for > 98% of the diversity (Figure 1). Of these, Firmicutes, Bacteroidetes, and Proteobacteria were relatively common in both upper GI (stomach and small intestine) and lower GI (cecum, large intestine, and fecal) samples, while Verrucomicrobia and Cyanobacteria were restricted mainly to the lower GI tract and Actinobacteria to the upper GI tract (Figure 1).

### 3.1 Diversity analyses

**Figure 2:**
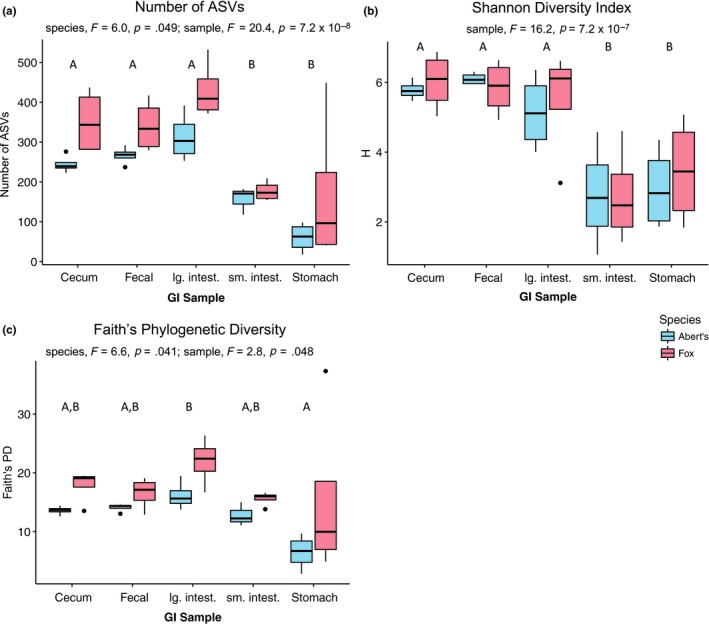
Results of *alpha*‐diversity analyses: (a) Number of ASVs, (b) Shannon's H, (c) Faith's Phylogenetic Diversity. Significant terms are listed for each metric. Samples grouped under different letters were significantly different (since there were no species × sample interactions, the values compared are averaged over species)

Comparisons of *alpha*‐diversity revealed no evidence of a species by GI sample interaction, but at least one of the main effects was significant for each *alph*a‐diversity metric (Figure 2). Specifically, GI sample location was significant in models for number of ASVs (linear mixed‐effects model, *F* = 20.4, *p* = 7.263 × 10^–8^; Figure 2a), Shannon's diversity (linear mixed‐effects model, *F* = 16.2, *p* = .048; Figure 2b), and Faith's phylogenetic diversity (linear mixed‐effects model, *F* = 2.8, *p* = 6.502 × 10^–7^; Figure 2c). In general, *alpha*‐diversity was higher in GI samples from the lower GI tract (cecum, large intestine, and fecal samples) than the upper GI tract (stomach and small intestine), although the only difference in Faith's phylogenetic diversity was that samples from the stomach had lower diversity than those from the large intestine (ls means with Tukey's HSD adjustment; Figure 2). There was also a significant species effect for number of ASVs (linear mixed‐effects model, *F* = 6.0, *p* = .049; Figure 2a) and Faith's phylogenetic diversity (linear mixed‐effects model, *F* = 6.6, *p* = .041; Figure 2c), with higher diversity detected in fox squirrels in both cases. 

**Figure 3:**
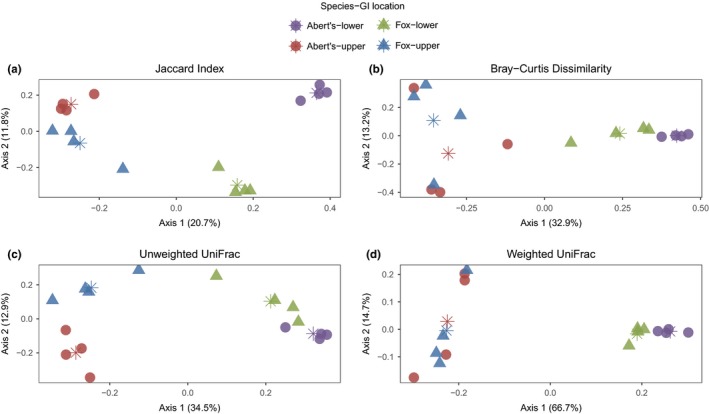
PCoA plot of *beta*‐diversity metrics for upper and lower GI tract samples from fox and Abert's squirrels: (a) Jaccard Index, (b) Bray‐Curtis dissimilarity, (c) Unweighted UniFrac, and (d) Weighted UniFrac. Stars represent centroids for each group

PERMANOVA analyses of all the *beta*‐diversity metrics revealed significant clustering of upper and lower GI tract samples in both species, suggesting differentiation of composition between upper and lower regions of the GI tract (Table 1; Figure 3; Figure S1 shows a PCoA plot of all sample locations from each individual). Furthermore, all metrics separated the lower GI tract samples between fox and Abert's squirrels (Table 1; Figure 3). Upper GI tract samples were differentiated between the species by the Jaccard index and unweighted UniFrac, but not by Bray–Curtis dissimilarity or weighted UniFrac (Table 1; Figure 3). These results suggest that lower and upper GI tract communities between the species are differentiated whether or not phylogenetic distances among community members are considered. Moreover, while lower GI tract communities are separable regardless of whether abundance estimates are considered, upper GI tract samples from the two species are differentiated primarily by taxa that were present in low abundance. Comparisons of within‐group dispersions also provided some evidence for differences in community variability between groups, particularly for the Jaccard index, where all comparisons were significant except for the comparison of upper GI samples between the species (Table 1). Inspection of PCoA plots indicates that in cases where within‐group dispersions were different, centroid locations of groups were also clearly separated, which suggests that both factors could have contributed to the significant PERMANOVA results (Figure 3). Notably, within‐group dispersion comparisons between the species for the lower GI samples were significant for three of the four tests, although only one was significant after correcting for multiple testing. Overall, these data demonstrate that upper and lower GI tract samples within each species harbor distinct microbial communities and also that communities differ between the species, particularly for lower GI tract samples. Furthermore, microbial communities in the lower GI tract of Abert's squirrels tend to be less variable than those of fox squirrels, though some of these differences were not significant after correcting for multiple testing.

**Table 1:** Results of beta‐group significance and beta‐group dispersion comparisons

Comparison	PERMANOVA pseudo‐F	PERMANOVA *p*‐value/*q*‐value	Betadisper pseudo‐F	Betadisper *p*‐value/*q*‐value
Jaccard index				
A‐lower versus A‐upper	3.55	**.030/.036**	4.07	**.011/.032**
F‐lower versus F‐upper	1.99	**.029/.036**	2.44	.053/.106
A‐lower versus F‐lower	2.85	**.029/.036**	4.44	**.005/.021**
A‐upper versus F‐upper	1.18	**.029/.036**	0.47	.645/.645
Bray‐Curtis				
A‐lower versus A‐upper	5.00	**.028/.036**	0.93	.366/1.00
F‐lower versus F‐upper	2.99	**.030/.036**	0.81	.436/1.00
A‐lower versus F‐lower	1.95	**.026/.036**	2.98	.031/.123
A‐upper versus F‐upper	0.79	.515/.515	0.31	.766/1.00
Unweighted UniFrac				
A‐lower versus A‐upper	7.09	**.027/.032**	3.97	**.010/.041**
F‐lower versus F‐upper	3.00	**.027/.032**	1.72	.137/.273
A‐lower versus F‐lower	2.40	**.031/.032**	2.38	.064/.192
A‐upper versus F‐upper	1.81	**.031/.032**	1.21	.270/.274
Weighted UniFrac				
A‐lower versus A‐upper	15.25	**.028/.037**	2.36	.092/.276
F‐lower versus F‐upper	11.16	**.029/.037**	2.32	.803/1.00
A‐lower versus F‐lower	1.91	**.030/.037**	2.36	.020/.080
A‐upper versus F‐upper	0.28	.911/.911	2.42	.501/1.00

Note

Tests significant after false‐discovery rate correction (*q*‐values) are in bold. A = Abert's; F = fox. Lower = lower GI tract (large intestine, cecum, and fecal); Upper = upper GI tract (stomach and small intestine).

### 3.2 Core microbiomes

The upper GI tract core microbiomes of fox and Abert's squirrels were small, including only two and three ASVs, respectively. The core microbiomes of both species included *Streptococcus gallolyticus* and *Lactobacillus salivarius*, with another *Lactobacillus* ASV being present in Abert's squirrels. Using a less stringent definition of the core microbiome requiring that a taxon is present in only 80% of samples rather than 100% increased the size of the core microbiomes to four and five ASVs, respectively, with three of those being shared between the species. The lower GI tract core microbiomes included much more diversity, with the Abert's squirrel core microbiome including more than double the number of ASVs compared to fox squirrels (68 vs. 26, respectively; Table S1). There was little overlap, with only five taxa shared between the species. The shared taxa include two ASVs from the family Lachnospiraceae and two from the Ruminococcaceae (both from the order Clostridiales) and one member of the Oxalobacteraceae family. Core taxa unique to fox squirrels were dominated by members of the families Ruminococcaceae and Lachnospiraceae, which were represented by nearly equal numbers of ASVs, with four additional families represented by one to two ASVs (Table S1). Core taxa unique to Abert's squirrels were also dominated by Lachnospiraceae and Ruminococcaceae, though diversity within these families was much higher relative to that found in fox squirrels with more than three times the number of representatives from Lachnospiraceae and double the number of representatives from Ruminococcaceae (Table S1). In addition, seven families were represented in the Abert's squirrel core microbiome that was not found in fox squirrel core microbiome, including multiple ASVs from Prevotellaceae (six ASVs) and Acidaminococcaceae (three ASVs).

### 3.3 Within species abundance comparisons between upper and lower GI tracts

**Figure 4:**
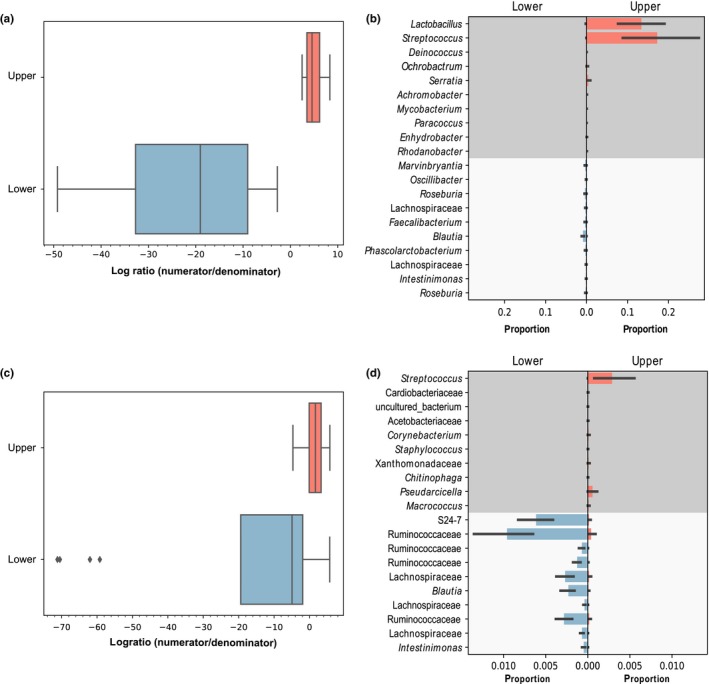
(a) Log ratios for balance y0 from Abert's upper and lower GI samples. (b) ASVs with the top 10 most positive and negative log ratios for balance y0 from Abert's upper and lower GI samples. Numerator taxa are in dark gray, while denominator taxa are in light gray. (c) Log ratios for balance y0 from fox upper and lower GI samples. (d) ASVs with the top 10 most positive and negative log ratios for balance y0 from fox upper and lower GI samples. Numerator taxa are in dark gray, while denominator taxa are in light gray

Differential abundance analysis with ANCOM identified 39 ASVs that were differentially abundant in Abert's squirrel upper and lower GI tracts (10 were higher in the upper tract and 29 were higher in the lower tract; Table 2). Hypergeometric tests revealed that ASVs with higher abundance in the Abert's upper GI tract included one enriched class (Lactobacillaceae, 51.7‐fold enrichment, *p* = 9.03E^−05^) and one enriched genus (*Lactobacillus*, 51.7‐fold enrichment, *p* = 9.03E^−05^; Table 2), while the lower tract included three enriched classes (Prevotellaceae, 7.1‐fold enrichment, Benjamini–Hochberg adjusted *p* = .0020; Bacteroidaceae, 8.9‐fold enrichment, Benjamini–Hochberg adjusted *p* = .0335; Alcaligenaceae, 10.2‐fold enrichment, Benjamini–Hochberg adjusted *p* = .0335) and five enriched genera (*Prevotella*, 10.2‐fold enrichment, Benjamini–Hochberg adjusted *p* = .0335; *Roseburia*, 6.5‐fold enrichment, Benjamini–Hochberg adjusted *p* = .0500; *Bacteroides*, 8.9‐fold enrichment, Benjamini–Hochberg adjusted *p* = .0335; *Parasutterella*, 23.8‐fold enrichment, Benjamini–Hochberg adjusted *p* = .0078; *Faecalibacterium*, 7.1‐fold enrichment, Benjamini–Hochberg adjusted *p* = .0462) (Table 2). Gneiss analysis revealed a significant difference between log ratios for the upper and lower GI samples for balances y0 – y8. We chose to further examine balance y0 since it had the largest effect size and was also the most basal partition, such that all other significant balances were subsets of y0 (balance y0: linear model, *t* = 7.81, Benjamini–Hochberg adjusted *p* = 4.38e^−7^; all tested balances shown in Figure S2). Specifically, the highly negative log ratio for the lower GI samples indicated that 272 denominator taxa were at higher abundance in the lower GI samples than were the 393 numerator taxa, whereas the opposite was true for the upper GI samples (Figure 4a). At the level of class, hypergeometric tests indicated that the denominator taxa were enriched for Lachnospiraceae (1.31‐fold enrichment, Benjamini–Hochberg adjusted *p* = .0014) and S24‐7 (1.76‐fold enrichment, Benjamini–Hochberg adjusted *p* = .0024), while no taxa were enriched at the level of genus. No taxa in the numerator were enriched at either taxonomic level. Figure 4b shows the top ten taxa with the most positive and negative log ratios, which includes several taxa that were identified as differentially abundant by ANCOM.

**Table 2:** Summary of ANCOM results for differentially abundant taxa between upper and lower GI tracts of Abert's and fox squirrels

Species	Abundance higher in upper GI tract (#ASVs)	Abundance higher in lower GI tract (#ASVs)
Abert's	Lactobacillaceae (3) *Lactobacillus* (3)* Streptococcaceae (1) *Streptococcus* (1) Brucellaceae (1) *Ochrobactrum* Staphylococcaceae (1) *Staphylococcus* (1) Xanthomonadales (1) Unclassified (1) Enterobacteriaceae (1) *Serratia* (1) Methylobacteriaceae (1) *Methylobacterium* (1) Sphingomonadales (1) SD04E11 (1)	Prevotellaceae (5)* *Prevotella* (2)* Unclassified (3)
		Lachnospiraceae (10) *Roseburia* (2)* *Marvinbryantia* (2) *Blautia* (2) *Acetitomaculum* (1) Unclassified (3)
		Bacteroidaceae (2)* *Bacteroides* (2)*
		Alcaligenaceae (2)* *Parasutterella* (2)*
		Ruminococcaceae (7) *Faecalibacterium* (2)* *Oscillibacter* (1) Unclassified (4)
		vadinHA64 (1) Unclassified (1)
		S24‐7 (1) Unclassified (1)
		Acidaminococcaceae (1) *Phascolarctobacterium* (1)
Fox	Streptococcaceae (2)* *Streptococcus* (2)* Lactobacillaceae (1) *Lactobacillus* (1) Pseudonocardiaceae (1) Methylobacteriaceae (1) *Methylobacterium* (1) Brucellaceae (1) *Ochrobactrum* (1) Moraxellaceae (1) *Enhydrobacter* (1) Caulobacteraceae (1) *Brevundimonas* (1) Rhodobacteraceae (1) *Stappia* (1)	Lachnospiraceae (6) *Blautia* (2) *Marvinbryantia* (1) *Acetitomaculum* (1) Unclassified (2)
		Ruminococcaceae (5) Unclassified (5)
		Alcaligenaceae (1) *Parasutterella* (1)
		S24‐7 (2) Unclassified (2)
		Bacteriodales (1) Unclassified (1)

Note

Taxa with an asterisk were enriched within each set.

In fox squirrels, ANCOM identified 24 ASVs that differed in abundance between upper and lower GI tracts (nine were higher in the upper tract and 15 were higher in the lower tract; Table 2). ASVs higher in abundance in the upper GI tract included one enriched class (Streptococcaceae, 39.7‐fold enrichment, Benjamini–Hochberg adjusted *p* = .0025) and one enriched genus (*Streptococcus*, 39.7‐fold enrichment, Benjamini–Hochberg adjusted *p* = .0025). No taxa were enriched in the set that was higher in abundance in the lower GI tract. Gneiss analysis revealed that balances y0–y9 were all significantly differentiated between upper and lower GI samples. Since balance y0 was the most basal partition and had the largest effect size, we chose to analyze it further (balance y0: linear model, *t* = 3.90, Benjamini–Hochberg adjusted *p* = .0022; all tested balances are shown in Figure S3). The negative log ratio for the lower GI tract indicated that the abundance of 308 denominator taxa was higher relative to the 812 numerator taxa in these samples, while upper GI samples had a higher abundance of numerator taxa (Figure 4c). At the level of class, hypergeometric tests indicated that the denominator taxa were enriched for Lachnospiraceae (1.67‐fold enrichment, Benjamini–Hochberg adjusted *p* = 1.85e^−14^), while nothing was enriched in the numerator taxa. At the genus level, *Coprococcus* (2.64‐fold enrichment, Benjamini–Hochberg adjusted *p* = .0021) and *Marvinbryantia* (2.29‐fold enrichment, Benjamini–Hochberg adjusted *p* = .0021) were both enriched in the denominator, while no taxa were enriched in the numerator set. Figure 4d shows taxa with the top 10 most positive and negative log ratios, which includes several taxa identified by ANCOM.

### 3.4 Between species abundance comparisons between upper and lower GI tracts

Differential abundance analysis with ANCOM did not identify any differences between fox and Abert's squirrels for the upper GI samples. For lower GI samples, 23 ASVs were more abundant in Abert's squirrels than fox squirrels, while 13 ASVs were more abundant in fox squirrels (Table 3). There was broad overlap between these sets of taxa at the level of class, with representatives from Lachnospiraceae, Ruminococcaceae, Prevotellaceae, S24–7, and Alcaligenaceae found in both sets (Table 3). While several taxonomic designations were enriched in the Abert's squirrel set, none were enriched in the fox squirrel set (Table 3). In Abert's squirrels, Lachnospiraceae (1.7‐fold enrichment, Benjamini–Hochberg adjusted *p* = .0328), Prevotellaceae (8.9‐fold enrichment, Benjamini–Hochberg adjusted *p* = .0002), and Alcaligenaceae (28.2‐fold enrichment, Benjamini–Hochberg adjusted *p* = .0029) were all enriched. Furthermore, within Lachnospiraceae the genus *Marvinbryantia* was enriched (5.0‐fold enrichment, Benjamini–Hochberg adjusted *p* = .0068), within Prevotellaceae the genus *Prevotella* was enriched (27.8‐fold enrichment, Benjamini–Hochberg adjusted *p* = .0019), and within Alcaligenaceae the genus *Parasutterella* was enriched (27.8‐fold enrichment, Benjamini–Hochberg adjusted *p* = .0019).

**Table 3:** Summary of ANCOM results for differentially abundant taxa between lower GI tracts of Abert's and fox squirrels

Abundance higher in Fox squirrel (# ASVs)	Abundance higher in Abert's squirrel (# ASVs)
Ruminococcaceae (5) Unclassified (5) Lachnospiraceae (3) *Marvinbryantia* (1) *Blautia* (1) Unclassified (1) S24‐7 (2) Unclassified (2) Bacteroidaceae (1) *Bacteroides* (1) Alcaligenaceae (1) *Parasutterella* Prevotellaceae (1) Unclassified (1)	Lachnospiraceae (11)* *Marvinbryantia* (4)* *Blautia* (2) *Dorea* (1) *Roseburia* (1) Unclassified (3) Prevotellaceae (5)* *Prevotella* (2)* Unclassified (3) Ruminococcaceae (3) *Oscillibacter* (1) Unclassified (2) Alcaligenaceae (2)* *Parasutterella* (2)* S24‐7 (1) Unclassified (1) Coriobacteriaceae (1) *Enterorhabdus* (1)

Note

Taxa with an asterisk were enriched within each set.

**Figure 5:**
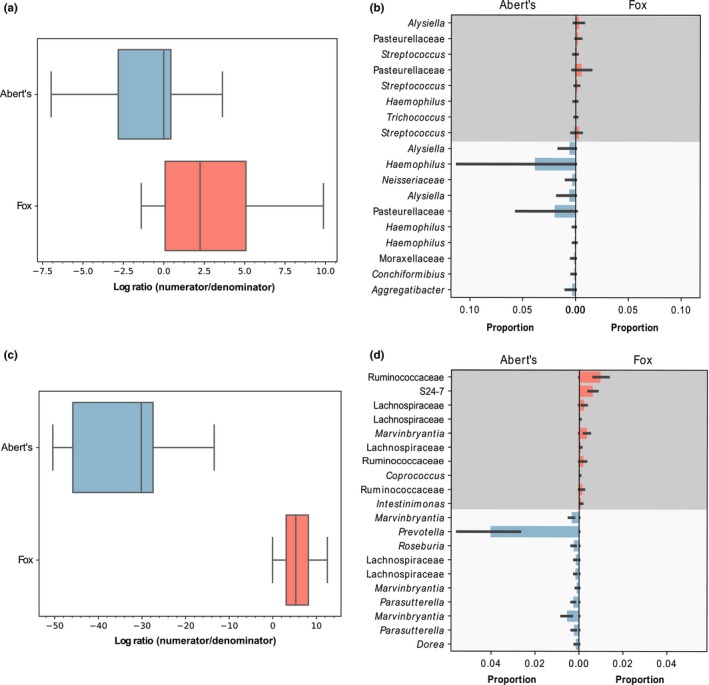
(a) Log ratios for balance y4 from comparisons of upper GI samples between fox and Abert's squirrels. (b) ASVs with the top 10 most positive and negative log ratios for balance y4 from comparisons of upper GI samples between fox and Abert's squirrels. Numerator taxa are in dark gray, while denominator taxa are in light gray (the numerator included only eight taxa). (c) Log ratios for balance y0 from comparisons of lower GI samples between fox and Abert's squirrels. (d) ASVs with the top 10 most positive and negative log ratios for balance y0 from comparisons of lower GI samples between fox and Abert's squirrels. Numerator taxa are in dark gray, while denominator taxa are in light gray

Gneiss revealed significant differences between the upper GI tract samples of fox and Abert's squirrels for balances y4 and y9. Since balance y4 had a larger effect size and balance y9 was a subset of balance y4, we chose to further analyze balance y4 (balance y4: linear mixed‐effects model, Benjamini–Hochberg adjusted *p* = .0254; all tested balances shown in Figure S4). For this balance, the negative log ratio for Abert's squirrel indicates that the 12 taxa in the denominator were at higher abundance relative to the eight taxa in the numerator, while the opposite was found for fox squirrels (Figure 5a). Figure 5b shows taxa with the most positive and the most negative log ratios and their relative abundance in each species. Due to the small number of taxa in the numerator and denominator we did not test for enrichment of particular taxa, but many ASVs from the same genus or class were present in both lists. For lower GI samples, only balance y0 was significantly different between species (balance y0: linear mixed‐effects model, Benjamini–Hochberg adjusted *p* = .0043; all tested balances are shown in Figure S5). This was the most basal partition, indicating a fundamentally different community composition in the lower GI tract of fox and Abert's squirrels. Specifically, the 222 denominator taxa were in higher abundance relative to the 1,007 numerator taxa in Abert's squirrels (indicated by the negative log ratio), while the opposite was true for fox squirrels (Figure 5c). Enrichment analyses of the communities representing the numerator and denominator at the level of class revealed no significant enrichment for the numerator community, but Lachnospiraceae (1.24‐fold enrichment, Benjamini–Hochberg adjusted *p* = .0497) and Prevotellaceae (2.29‐fold enrichment, Benjamini–Hochberg adjusted *p* = .0470) were enriched in the community representing the denominator. Several ASVs that were identified as differentially abundant by ANCOM were present in the list of the top 10 most negative log ratios including representatives from *Marvinbryantia*, *Prevotella*, and *Parasutterella* (Figure 5d).

## 4 DISCUSSION

Within species comparisons of samples from different locations along the GI tract revealed several patterns that are generally consistent with previous studies comparing microbial communities along the length of the GI tract. First, *beta‐*diversity analyses clearly separated samples into two major clusters representing the upper (stomach and small intestine) and lower (large intestine, cecum, and fecal) GI tract, with much lower differentiation among samples within these regions. Furthermore, most *alpha*‐diversity measurements were higher in samples from the lower GI tract than the upper GI tract. These results parallel findings in several other rodent species where samples from the upper and lower GI tract are clearly delineated by community membership, and lower GI samples typically have higher diversity (Gu et al., 2013; Kohl, Dearing, et al., 2017; Lu et al., 2015; Suzuki & Nachman, 2016). Consistent with lower overall diversity in the upper GI tract, the core microbiota of upper GI samples were comprised of few species, which also indicates high individual heterogeneity in this community. The upper core microbiota of both species included ASVs representing *Lactobacillus* and *Streptococccus*, which are both facultative anaerobes capable of persisting in the more oxygen‐rich environment of the upper GI tract. In contrast, the core microbiota from the lower GI tract in both species was more diverse, mainly comprised of fermentative polysaccharide‐degrading anaerobes (e.g., from families Ruminoccocaceae and Lachnospiraceae), which is expected given that these regions of the GI tract are where fermentation of plant‐based materials occurs in hindgut fermenters. Differences between communities from the upper and lower GI tract were also reinforced by the results of differential abundance analyses, which identified differentially abundant facultative anaerobes in the upper GI tract samples and mostly fermentative polysaccharide‐degrading anaerobes in the lower GI tract of both species.

Despite living in the same habitat, comparisons between fox and Abert's squirrels revealed considerable divergence in their microbiota. Abert's squirrels have an unusual diet that is high in fiber (Keith, 1965; Snyder, 1992; States & Wettstein, 1998). This diet has likely shaped the evolution of the Abert's squirrel GI tract, as evidenced by morphological adaptations, particularly in the lower GI tract, that increase the surface area for nutrient absorption and decrease transit time (Murphy & Linhart, 1999). Consistent with these observations, the microbial communities of the fox and Abert's squirrel lower GI tracts were clearly delineated by all *beta*‐diversity analyses. Moreover, differential abundance analyses suggest that the Abert's squirrel microbiota is enriched for microbes known to be associated with the degradation of plant fiber (primarily members of families Prevotellaceae and Lachnospiraceae). For example, Abert's squirrel lower GI tracts had higher diversity and abundance of several representatives from the family Prevotellaceae, with five ASVs present in the lower core microbiome (compared with none in fox squirrels), which were all present in higher abundance in Abert's squirrels (one additional representative was at higher abundance in fox squirrels). *Prevotella* has been repeatedly associated with high‐fiber diets in human studies (De Filippo et al., 2010; Schnorr et al., 2014; Yatsunenko et al., 2012) and is also a major constituent of the microbiome of ruminants and other herbivorous mammals (AvguÅ¡tin, Flint, & Whitehead, 1992; Flint, Scott, Duncan, Louis, & Forano, 2012; Gruninger, McAllister, & Forster, 2016; Kohl, Varner, Wilkening, & Dearing, 2018; Li, Li, Beasley, et al., 2016; Li, Li, Yao, et al., 2016). Within the family Lachnospiraceae, the lower GI tract of Abert's squirrels had higher abundance and diversity within the genus *Marvinbryantia*, with four ASVs represented in the lower core microbiome (compared to one in fox squirrels), all of which were in higher abundance in Abert's squirrels relative to fox squirrels. The one currently described species in this genus, *Marvinbryantia formatexigens*, is known to have cellulolytic capabilities (Levin et al., 2015). Thus, the presence of several undescribed representatives of this genus in the lower GI tract of Abert's squirrels highlights potential involvement in facilitating digestion of the high‐fiber diet of Abert's squirrels. Although differential abundance analysis indicated that more ASVs within the fibrolytic family Ruminococcaceae (Flint et al., 2012) were at higher abundance in fox than in Abert's squirrels, a few specific ASVs within this group were higher in Abert's squirrels. Moreover, the lower core microbiota of Abert's squirrels also included nearly double the number of representatives from the family Ruminococcaceae compared to fox squirrels, which suggests these taxa are stable members of the micro‐organismal community within the lower GI tract of Abert's squirrels. Altogether, these results suggest that expansions in diversity and increases in abundance of several key taxa in the lower GI tract of Abert's squirrels, particularly in the Prevotellaceae and Lachnospiraceae, may facilitate the intake and digestion of the high‐fiber diet of this species. We also note that since the current study focused only on Bacteria and Archaea, future metagenomic sequencing might reveal additional microorganisms (e.g., fungi or protozoa) that further facilitate the intake and processing of fiber‐rich food items.

Our analysis also revealed enrichment of taxa in Abert's lower GI tract that is unlikely to be involved in the direct breakdown of plant‐based fiber. For example, the Abert's lower GI tract was enriched for *Parasutterella*, and multiple members of *Phascolarctobacterium* were present in the Abert's core microbiota while none were included in the fox squirrel core microbiota. While relatively little is known about these genera, cultured strains representing both genera are asaccharolytic, suggesting that they do not play a direct role in the breakdown of resistant plant fibers. However, cultured strains of *Phascolarctobacterium* require succinate as an energy source, which is produced in abundance by many saccharolytic bacteria in animal GI tracts. Thus, the increased presence of this genus, and perhaps other asaccharolytic bacteria, in the Abert's lower GI tract may be an indirect consequence of their high‐fiber diet.

In contrast to major differences between the microbial communities inhabiting the lower GI tract of fox and Abert's squirrels, the communities of the upper GI tracts were more similar. While these communities were differentiated by some *beta*‐diversity metrics, this was primarily driven by taxa present in low abundance. Furthermore, the upper core microbiomes were nearly identical, with Abert's squirrels having only one ASV that was not also present in the fox upper core microbiome, and there were no differentially abundant taxa identified. These results are concordant with morphological comparisons between the species, which showed considerably more morphological divergence between the lower GI tract of fox and Abert's squirrels relative to the upper GI tract. These morphological differences were assumed to reflect selection pressures to retain food for longer in the fermentation chambers of Abert's squirrels given their high‐fiber, low‐quality diet (Murphy & Linhart, 1999). The fact that the relative amount of divergence in microbiota across the length of the GI tract in fox and Abert's squirrels mirrors these previously established morphological patterns further reinforces the hypothesis that the lower GI tract of Abert's squirrels in particular has evolved to suit their unique diet. Moreover, our findings suggest that community composition of the lower GI tract may be influenced more by variation in diet than that of the upper GI tract. Although additional comparative studies of microbiota across the length of the GI tract in species with different diets are necessary to confirm whether this is a general pattern across taxa, at least one previous study also found that divergence in the microbiota between species increases moving from the upper to lower GI tract (Kohl, Dearing, et al., 2017).

Our analysis shows that overall *alpha*‐diversity was generally higher in fox than in Abert's squirrels. The only exception to this was for Shannon's diversity metric (no species differences), which suggests that the more taxonomically diverse community of fox squirrels is unevenly distributed. Despite lower overall diversity in the Abert's squirrel microbiota, the core microbiota of Abert's squirrels was more diverse than that of fox squirrels and there was less individual to individual variation in the microbial community among Abert's squirrels compared than fox squirrels. We hypothesize that these disparities may be related to differences in diet breadth between the species. Specifically, the diet of Abert's squirrels may be more specialized than that of fox squirrels, particularly during winter when previous studies have shown that they feed on a relatively narrow range of food items (Allred, 2010; Hall, 1981; Keith, 1965; States & Wettstein, 1998). A narrower diet breadth may decrease individual‐level variability in gut microbiota (as observed for Abert's squirrels), since individuals consume similar diets. Wider diet breadth may lead to higher *alpha*‐diversity (as observed in fox squirrels) because of more opportunities for colonization by diverse microbes and an increased range of metabolic niches available (Heiman & Greenway, 2016; Laparra & Sanz, 2010; Reese & Dunn, 2018). Despite these predictions, comparisons of specialists and generalists within and between species have produced equivocal results (Blankenchip, Michels, Braker, & Goffredi, 2018; Lau et al., 2018; Li, Li, Beasley, et al., 2016; Reese & Dunn, 2018). Since we did not collect specific data on diet breadth of fox and Abert's squirrels, additional studies are necessary to fully test this hypothesis. Interestingly, gut length has also been recognized as a potential driver of variation in gut microbial diversity, with longer guts predicted to harbor more diversity (Reese & Dunn, 2018). Our results are inconsistent with this hypothesis as previous studies have demonstrated that the Abert's squirrel GI tract is considerably longer than that of fox squirrels (Murphy & Linhart, 1999).

While this study provides insight into factors that may play a role in shaping gut microbial diversity, we also note some limitations. Most importantly, our conclusions are based on a relatively small number of female animals captured during a narrow time window. Previous studies in wild squirrel populations have shown that gut microbial communities differ between the sexes and vary seasonally (Ren et al., 2017). Thus, additional sampling from both sexes, along with dietary analysis, would help to more rigorously evaluate hypotheses that we propose. Despite these limitations, our findings strengthen previous assertions that the high‐fiber diet of Abert's squirrels has shaped the evolution of the GI tract of this species (Murphy & Linhart, 1999). Moreover, our data corroborate previous research documenting distinct microbial communities in the upper and lower GI tract (Gu et al., 2013; Kohl, Dearing, et al., 2017; Lu et al., 2015; Suzuki & Nachman, 2016). Although few studies have compared microbial communities along the length of the GI tract in different species, our findings align with previous research suggesting the microbiota of the lower GI tract may be more divergent between species than microbial communities inhabiting the upper GI tract (Kohl, Dearing, et al., 2017).

## CONFLICT OF INTEREST

None declared.

## AUTHOR CONTRIBUTIONS

JMB, JCP, HKP, and AR designed the study; AR performed the research; JMB, AR, and CG analyzed the data, all authors helped write the paper.

## Data Availability

Demultiplexed sequencing reads are archived on the Short Read Archive (SRA) under accession number PRJNA574162. The feature table generated in Qiime 2 and a file with sequences of all ASVs is archived in the Dryad repository: https://doi.org/10.5061/dryad.931zcrjfn
