# Supplementary figures and images for: Comparative analysis of microbiota along the length of the gastrointestinal tract of two tree squirrel species (Sciurus aberti and S. niger) living in sympatry
Source: Ecol Evol. 2019 Nov 11;9(23):13344–58. doi: 10.1002/ece3.5789 (PMC6912893; doi:10.1002/ece3.5789)

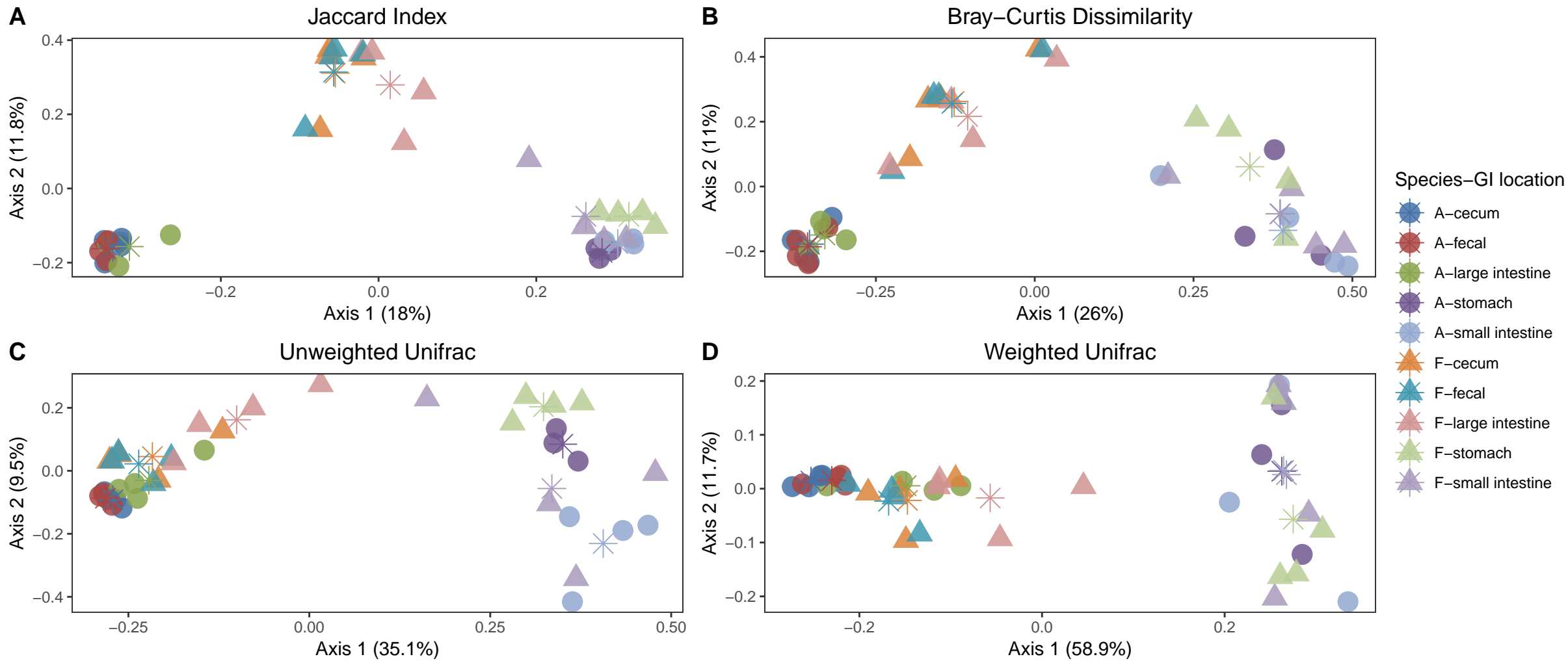

Supplement: Supplementary file 1 [file ECE3-9-13344-s001.pdf]

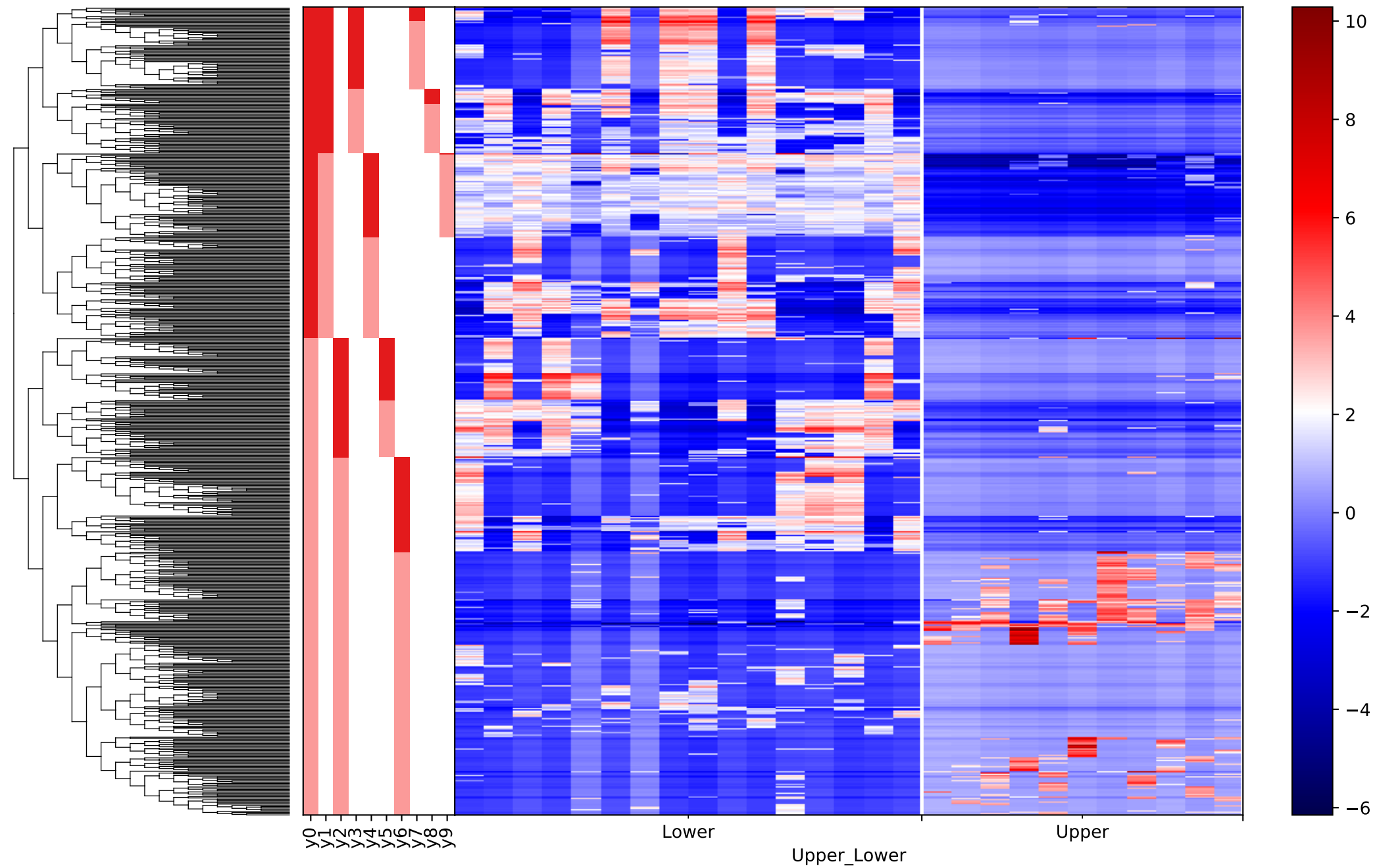

Supplement: Supplementary file 2 [file ECE3-9-13344-s002.pdf]

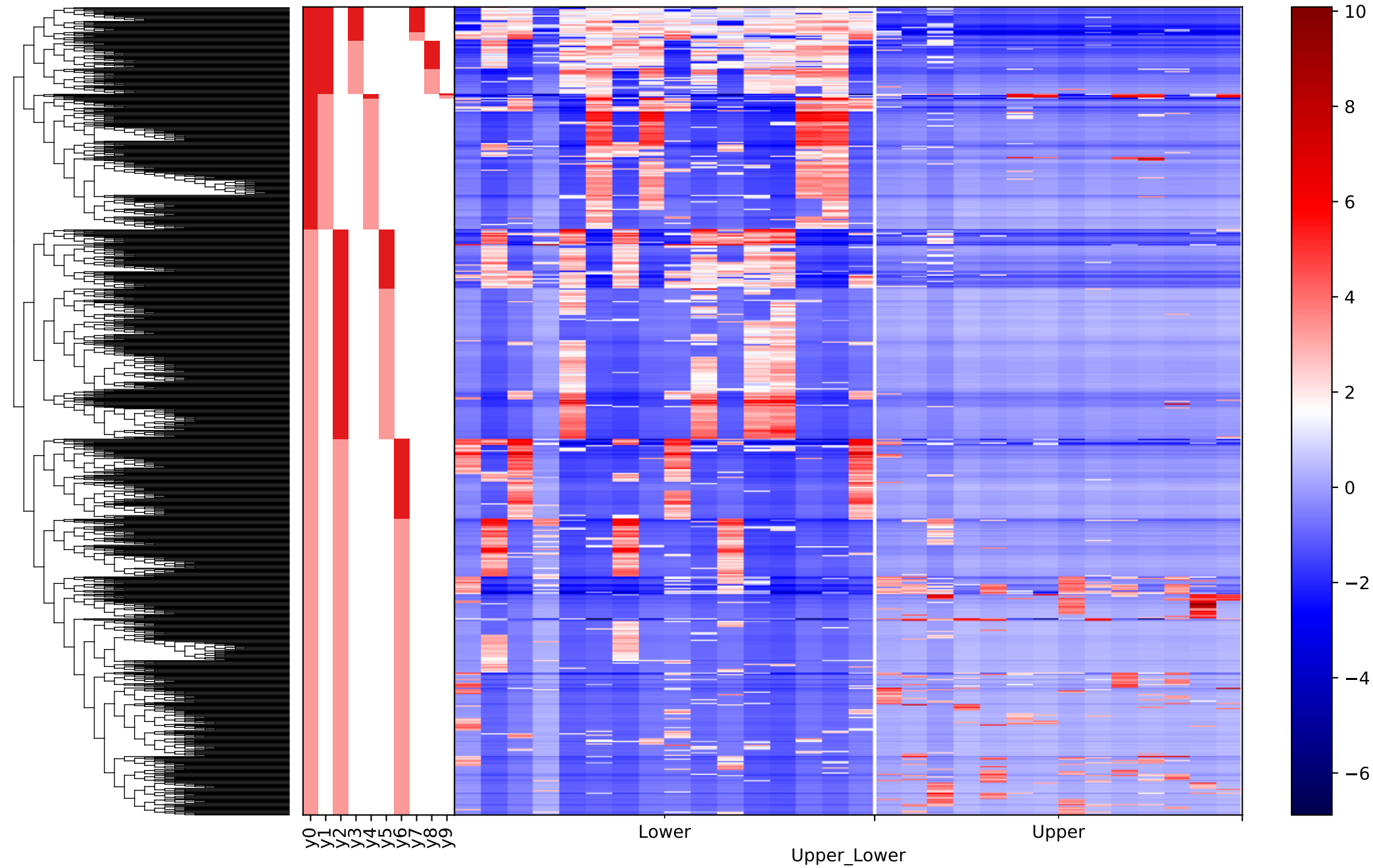

Supplement: Supplementary file 3 [file ECE3-9-13344-s003.pdf]

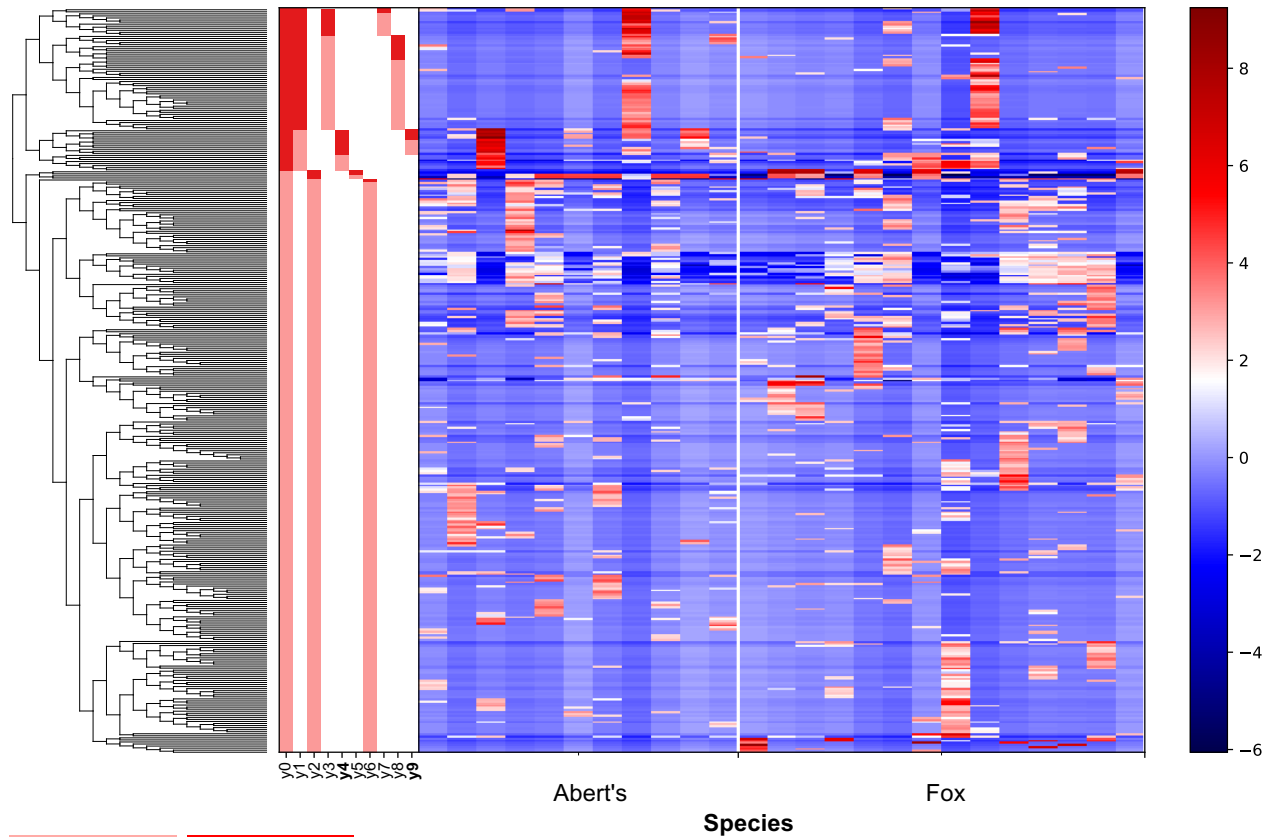

NUMERATOR

DENOMINATOR

Supplement: Supplementary file 4 [file ECE3-9-13344-s004.pdf]

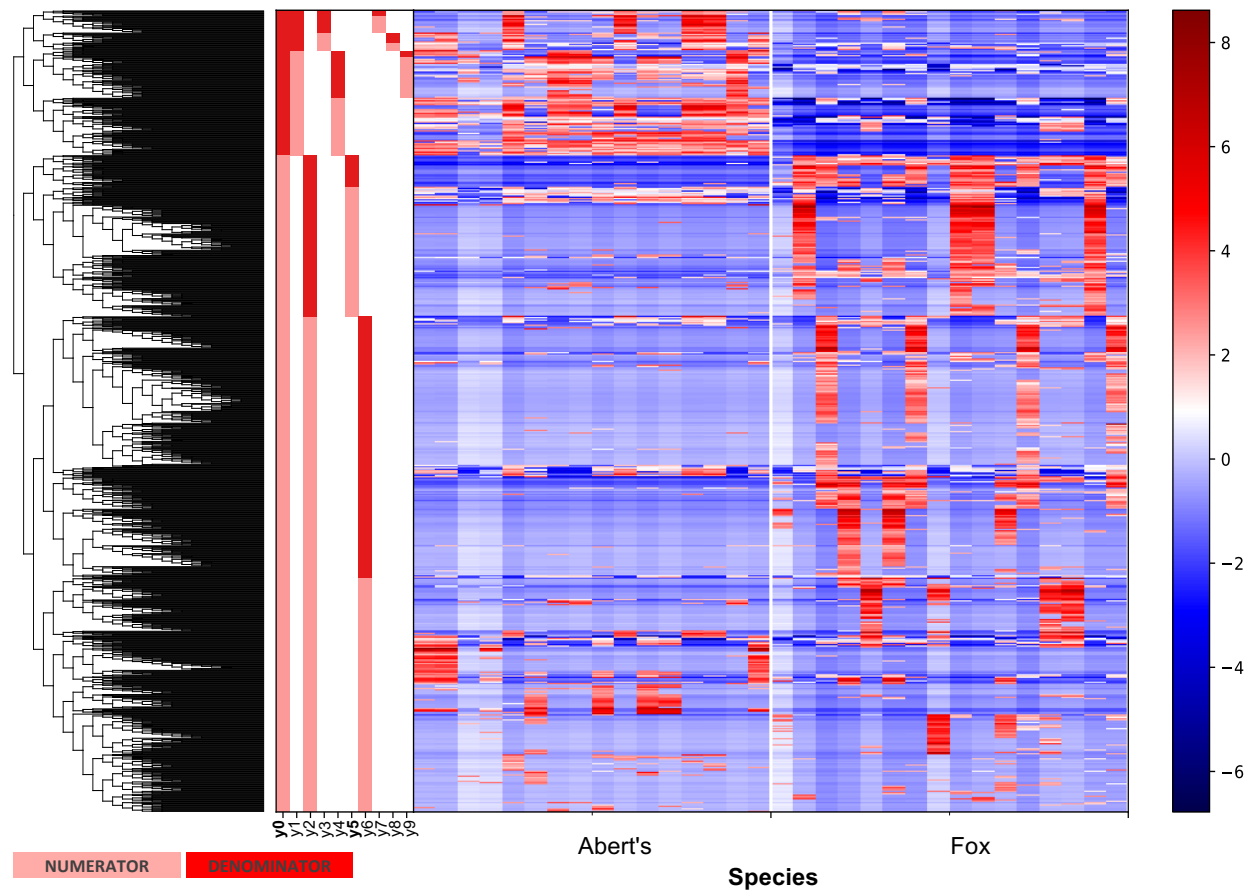

Supplement: Supplementary file 5 [file ECE3-9-13344-s005.pdf]
